# Combined Effects of Nutrients × Water × Light on Metabolite Composition in Tomato Fruits (*Solanum Lycopersicum* L.)

**DOI:** 10.3390/plants10071437

**Published:** 2021-07-14

**Authors:** Yangmin X. Kim, Suyoung Son, Seulbi Lee, Eunsung Jung, Yejin Lee, Jwakyung Sung, Choonghwan Lee

**Affiliations:** 1 National Institute of Agricultural Sciences, Rural Development Administration, Wanju 55365, Korea; yangmink@korea.kr (Y.X.K.); seulvi23@korea.kr (S.L.); leeyj418@korea.kr (Y.L.); 2 Department of Bioscience and Biotechnology, Konkuk University, Seoul 05029, Korea; syson119@naver.com; 3 Department of Systems Biotechnology, Konkuk University, Seoul 05029, Korea; jes708@naver.com; 4 Department of Crop Science, College of Agriculture, Life and Environment Sciences, Chungbuk National University, Cheongju 28644, Korea; 5 Research Institute for Bioactive-Metabolome Network, Konkuk University, Seoul 05029, Korea

**Keywords:** fruit quality, lycopene, metabolite profiling, supplemental red light, nitrogen

## Abstract

Tomato cultivation in the greenhouse can be facilitated by supplemental light. We compared the combined effects of nutrients, water, and supplemental light (red) on tomato fruit quality. To do this, three different nutrient conditions were tested, i.e., (1) low N, (2) standard N, and (3) high N. Water was supplied either at −30 kPa (sufficient) or −80 kPa (limited) of soil water potential. Supplemental red LED light was turned either on or off. The metabolites from tomato fruits were profiled using non-targeted mass spectrometry (MS)-based metabolomic approaches. The lycopene content was highest in the condition of high N and limited water in the absence of supplemental light. In the absence of red lighting, the lycopene contents were greatly affected by nutrient and water conditions. Under the red lighting, the nutrient and water conditions did not play an important role in enhancing lycopene content. Lower N resulted in low amino acids. Low N was also likely to enhance some soluble carbohydrates. Interestingly, the combination of low N and red light led to a significant increase in sucrose, maltose, and flavonoids. In high N soil, red light increased a majority of amino acids, including aspartic acid and GABA, and sugars. However, it decreased most of the secondary metabolites such as phenylpropanoids, polyamines, and alkaloids. The water supply effect was minor. We demonstrated that different nutrient conditions of soil resulted in a difference in metabolic composition in tomato fruits and the effect of red light was variable depending on nutrient conditions.

## 1. Introduction

Many recent research pieces have applied LED light to compensate for the shortage of light [1,2,3,4,5,6]. It has been known that the fruit yield and the chemical composition of fruit were affected by the light quantity and quality [7]. Recently, the effect of the spectra and the period of supplemental lighting on the production and quality of tomato fruits was investigated [1]. Among different colors of supplemental lighting, red supplemental light is known to increase tomato yields [8,9]. Red light is also considered the most efficient light for driving photosynthesis [4,10]. Light and water are compounds required for photosynthesis, and nutrients are essential for crop growth; therefore, changing the light supply might require changing the water and nutrient supply regime to produce good quality fruits because of their interactions [7].

In terms of regulating nutrient and water supply in horticulture, minimal nutrient and water use have been used to enhance crop quality [11]. Especially for tomato fruits, either low or medium-level nitrogen (N) supply was reported to be needed for the highest lycopene content in hydroponic culture and field conditions [12,13]. Low N supply was increasing the lycopene of tomato fruits grown in excess Mg soil [14]. On the contrary, a high level of NPK fertilizer supply was enhancing the tomato fruits’ lycopene content in the field experiment [15]. Deficit irrigation improves the tomato fruit quality by increasing fruit soluble solids levels and concentrations of hexoses, citric acid, and potassium [16]. 

In the current study, we tested whether or not imposing various nutrients (low N, standard N, and high N) and water supply (limited vs. sufficient) either with or without supplemental red LED light would improve the fruit quality of tomato. To establish the three different nutrient conditions, we employed infertile soil and excessively fertile soil. Low N was achieved by no additional nitrogen fertilizer application for infertile soil, and the standard N was by standard nutrient supply for infertile soil. High N was the standard supply for excessively fertile soil. We tried to figure out if water and supplemental light conditions would influence the tomato fruit quality in soils with different nutrient conditions. Quantitative lycopene content and primary and secondary metabolites using non-targeted metabolomics were investigated for tomato fruits. Metabolomics is a comprehensive approach to evaluate different metabolomes under a specific set of conditions [17]. We have applied it for various crops in response to varying environmental factors, including light and mineral nutrient supply [1,14,18,19,20].

## 2. Results

### 2.1. Lycopene Contents of Tomato Fruits Cultivated under Different Mineral Nutrient, Water, and Light Condition

**Figure 1:**
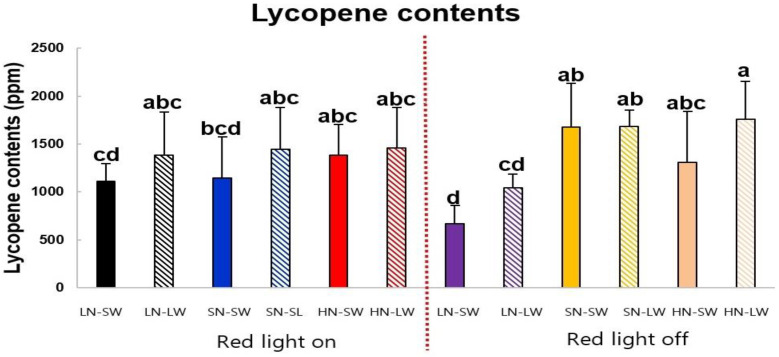
Lycopene contents of tomato fruits are influenced by nutrient, water, and light condition on a dry weight basis. LN, Low N; SW, sufficient water; LW, limited water; SN, standard N; HN, high N. Closed bars are sufficient water supply, and hatched bars are limited water supply. Mean values and standard deviations are presented (N = 5). The alphabetical letters above the bars mean significant differences in values by different nutrient × water × light conditions and they were determined by ANOVA followed by Duncan’s multiple range test (*p* < 0.05).

We measured the difference in lycopene contents in tomato fruits by combining nitrogen, water, and red light (Figure 1). In red light, lycopene content was rather constant in response to nutrient and water conditions. Therefore, the red light stabilized lycopene content through the nutrient and water regime. By contrast, in the absence of red light, lycopene content seemed to be strongly affected by N condition; low N resulted in lower lycopene contents than standard and high N. High N × limited water condition might be one of practical strategies to enhance lycopene content as tomato is growing in the absence of supplemental red light. 

### 2.2. Non-Targeted Metabolite Profiling of Tomato Fruits Cultivated under Different Mineral Nutrient, Water, and Light Condition

**Figure 2:**
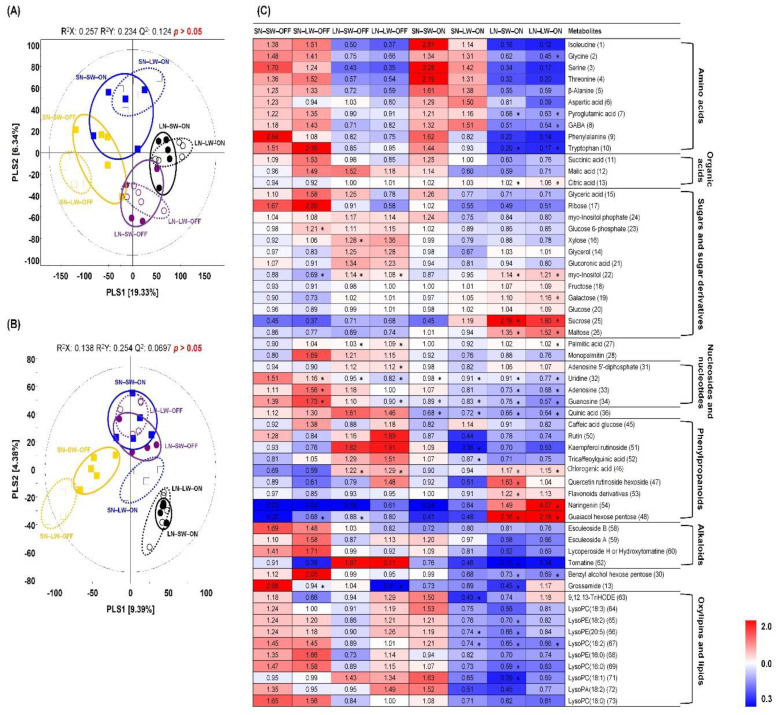
PLS-DA score plots from GC-TOF-MS (**A**) and UHPLC-LTQ-Orbitrap-MS (**B**) analysis of tomato cultivated under different nutrient, water, and light condition. Symbol, SN-SW-OFF (■); SN-LW-OFF (□); LN-SW-OFF (●); LN-LW-OFF (○); SN-SW-ON (■); SN-LW-ON (□); LN-SW-ON (●); LN-LW-ON (○). SN, standard N; SW, sufficient water; OFF, red light off; LN, Low N; LW, limited water; ON, red light on. Heat map analysis for the relative abundance of discriminant metabolites (VIP > 1.0) based on GC-TOF-MS and UHPLC-LTQ-Orbitrap-MS datasets (**C**). The colored squares (blue to red) indicate fold changes normalized by the average of each metabolite. * Significantly difference metabolite from control (SN-SW-OFF) evaluated by *t*-test (*p* < 0.05).

To investigate the effect of nutrient, water condition, and supplemental red light condition, we performed comprehensive primary and secondary metabolite profiling of tomato fruits using gas chromatography-time of flight-mass spectrometry (GC-TOF-MS) and ultrahigh performance liquid chromatography-linear trap quadrupole-orbitrap-mass spectrometry (UHPLC-LTQ-Orbitrap-MS). The PLS-DA score plot derived from GC-TOF-MS datasets displayed a distinct pattern between red light-on/-off and nutrient conditions by PLS1 (19.33%) and PLS2 (6.34%) (Figure 2A). We also observed a similar pattern in PCA plots, although the differences between groups were not clear compared to the PLS-DA plots (Figure S1A). Discriminant primary metabolites were identified and selected using variable importance in the projection value (VIP > 1.0) from PLS-DA models. A total of 37 primary metabolites containing ten amino acids, three organic acids, 13 sugars and sugar derivatives, two fatty acids and lipids, four nucleosides and nucleotides, one other, and four unknowns were characterized (Table S3). The relative levels of primary metabolites under different mineral nutrient, water, and light conditions were shown in a heatmap analysis and translated into fold-change values (Figure 2C). Most of the amino acids were abundant in red light-on and -off groups under standard nutrient conditions. Moreover, in red light-off groups under standard nutrient conditions, the level of 3 nucleosides, including uridine, adenosine, and guanosine, and quinic acid, were higher than other different conditions. Some sugars and sugar derivatives such as Myo-inositol, fructose, galactose, glucose, sucrose, and maltose showed higher levels in the red light-on group under low N conditions than other conditions. Similarly, sugars and sugar derivatives such as myo-Inositol phosphate, glucose 6-phosphate, xylose, glycerol, glucuronic acid, 2 fatty acids and lipids, ADP, and quinic acid were observed higher in LED-off groups under low N conditions. The UHPLC-LTQ-Orbitrap-MS (negative ion mode) data were used to examine differences among different conditions for secondary metabolites. The PLS-DA score plots showed that red light-off groups under standard nutrient conditions were distinguished from other conditions by PLS1 (9.39%) (Figure 2B). A total of 29 secondary metabolites, including 9 phenylpropanoids, 4 alkaloids, 10 oxylipins and lipids, 2 others, and 4 unknowns, were identified and selected as discriminant metabolites based on PLS-DA model VIP (>1.0) value (Table S4). The relative levels of discriminant metabolites were shown in the heat map (Figure 2C). According to the heat map analysis, alkaloids except for tomatine and most oxylipins and lipids were relatively high in red light-off groups under standard nutrient conditions. Moreover, low N, limited water and red light off condition (LN-LW-OFF) and standard nutrient, sufficient water, and red light on condition (SN-SW-ON) also showed higher oxylipins and lipids than other conditions. Phenylpropanoids were relatively high in red light on and off groups under low N conditions. 

**Figure 3:**
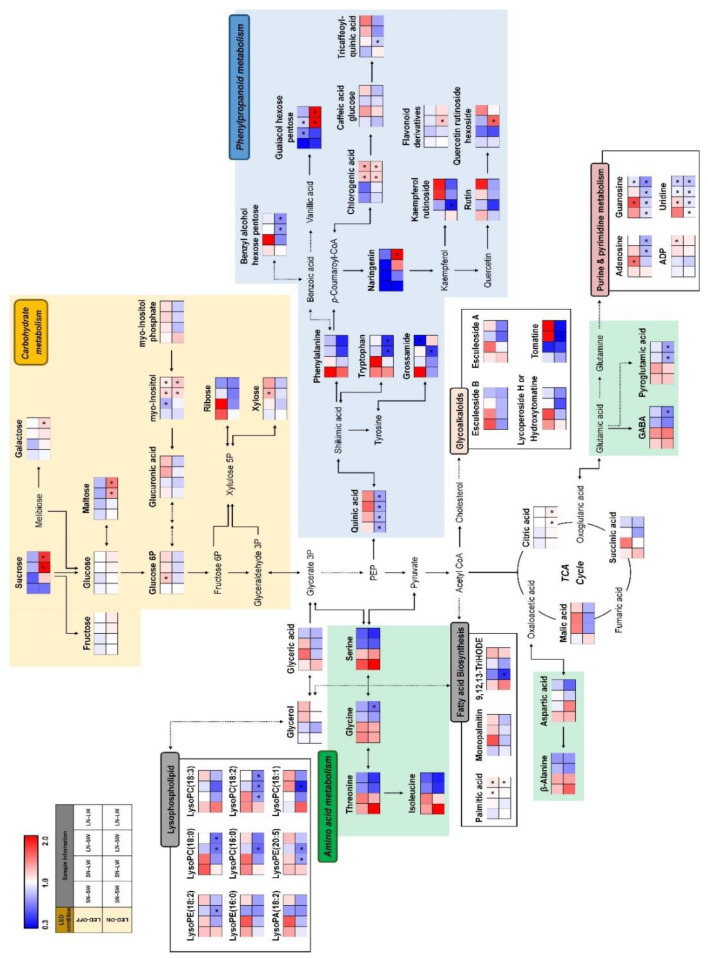
Constructed metabolic pathway for relative metabolite levels of tomato cultivated under different nutrient, water, and light condition. The metabolic pathway was modified from the KEGG pathway database (https://www.genome.jp/kegg/pathway.html; URL accessed on 4 June 2021). The colored squares (blue to red) indicate fold changes normalized by the average of each metabolite. * Significantly difference metabolite from control (SN-SW-OFF) evaluated by *t*-test (*p* < 0.05).

Moreover, we observed a metabolic pathway to understanding the distribution and the relationship between metabolites and different cultivation conditions (Figure 3). The metabolic pathway related to amino acid biosynthesis were upregulated in red light-on and -off groups under standard N conditions. Intriguingly, the lipids-related metabolism were highly expressed in red light-on and -off groups under standard N condition except for SN-LW-ON condition. Most glycoalkaloids and purine and pyrimidine metabolism-related metabolites, including esculeoside A, esculeoside B, hydroxytomatine, adenosine, guanosine, and uridine, were highly expressed in the red light-off group under standard N soil conditions. In contrast, carbohydrates and phenylpropanoids metabolism were upregulated in the red light -on and -off group under low N conditions. Collectively, different nutrient and lighting conditions can contribute to difference in levels of metabolite.

### 2.3. Non-Targeted Metabolite Profiling of Tomato Fruits Cultivated under Different Water and Light Condition for High N Soil

**Figure 4:**
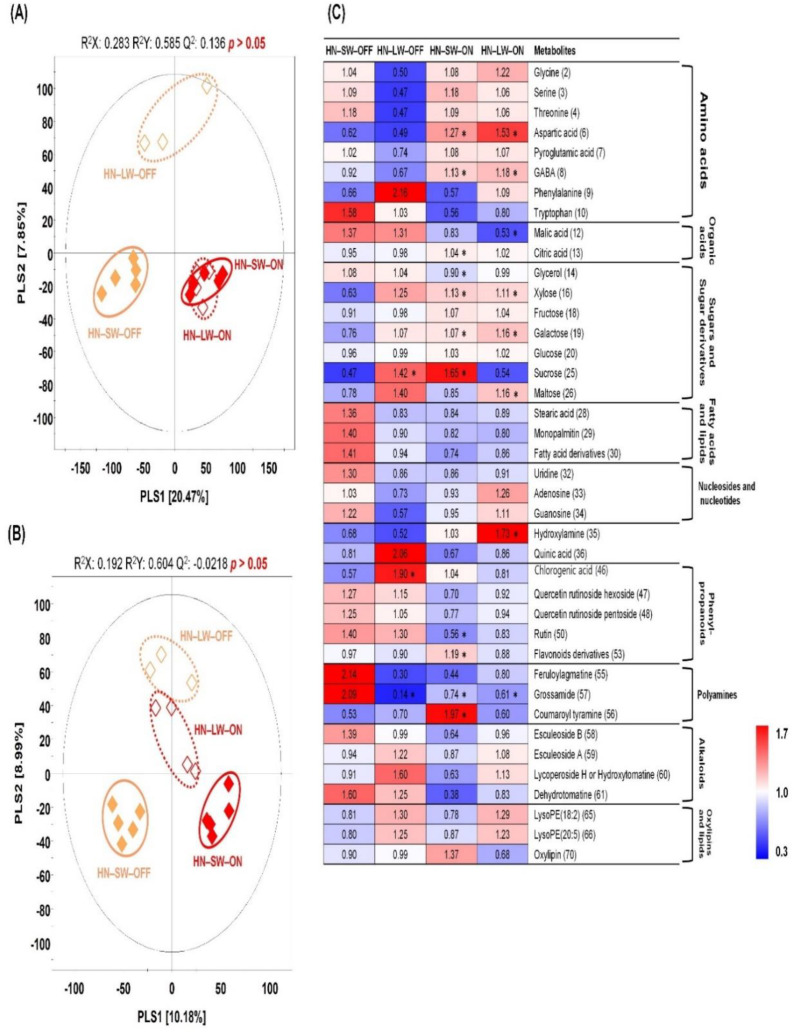
PLS-DA score plots from GC-TOF-MS (**A**) and UHPLC-LTQ-Orbitrap-MS (**B**) analysis of tomato cultivated under different water and light condition for high N soil. Symbol, HN-SW-OFF (◆); HN-LW-OFF (◇); HN-SW-ON (◆); HN-LW-ON (◇). HN, high N; SW, sufficient water; OFF, red light off; LW, limited water; ON, red light on. Heat map analysis for the relative abundance of discriminant metabolites (VIP > 1.0) based on GC-TOF-MS and UHPLC-LTQ-Orbitrap-MS datasets (**C**). The colored squares (blue to red) indicate fold changes normalized by the average of each metabolite. * Significantly difference metabolite from control (HN-SW-OFF) evaluated by *t*-test (*p* < 0.05).

In order to examine metabolites in tomato fruits cultivated with different water and red light condition under excessively fertile soil, we performed primary and secondary metabolite analysis in the same way as Result 2.3. The PLS-DA score plot based on GC-TOF-MS showed that red light-on groups were distinguished from red light-off groups by PLS1 (20.47%; Figure 4A). Unlike GC-TOF-MS analysis, PLS-DA score plots derived from UHPLC-LTQ-Orbitrap-MS revealed that red light-on/-off groups also showed distinct patterns by PLS1 (10.18%), and limited water conditions were discriminated from sufficient water condition by PLS2 (8.99%; Figure 4B). A total of 51 discriminated metabolites were selected and identified, of which GC-TOF-MS and UHPLC-LTQ-Orbitrap-MS identified 31 and 20 metabolites in the respective analytical instruments (Table S4). These identified metabolites are visualized in a heatmap (Figure 4C). Red lighting increased many amino acids and sugars. Most amino acids, organic acids, sugars and sugar derivatives, and fatty acids and lipids levels showed similar patterns in red light-on groups. However, red light-off groups indicated different patterns depending on water conditions. 3 Fatty acids and lipids and three nucleosides were higher in sufficient water conditions than in limited water conditions. For secondary metabolites, red lighting decreased phenylpropanoids, polyamines, and dehydrotomatine compared to without red lighting. In HN-LW-OFF and HN-LW-ON groups, the level of alkaloids and lipids showed a similar pattern. Particularly, feruloylagmatine and grossamide (polyamines) showed the highest level in the HN-SW-OFF group.

**Figure 5:**
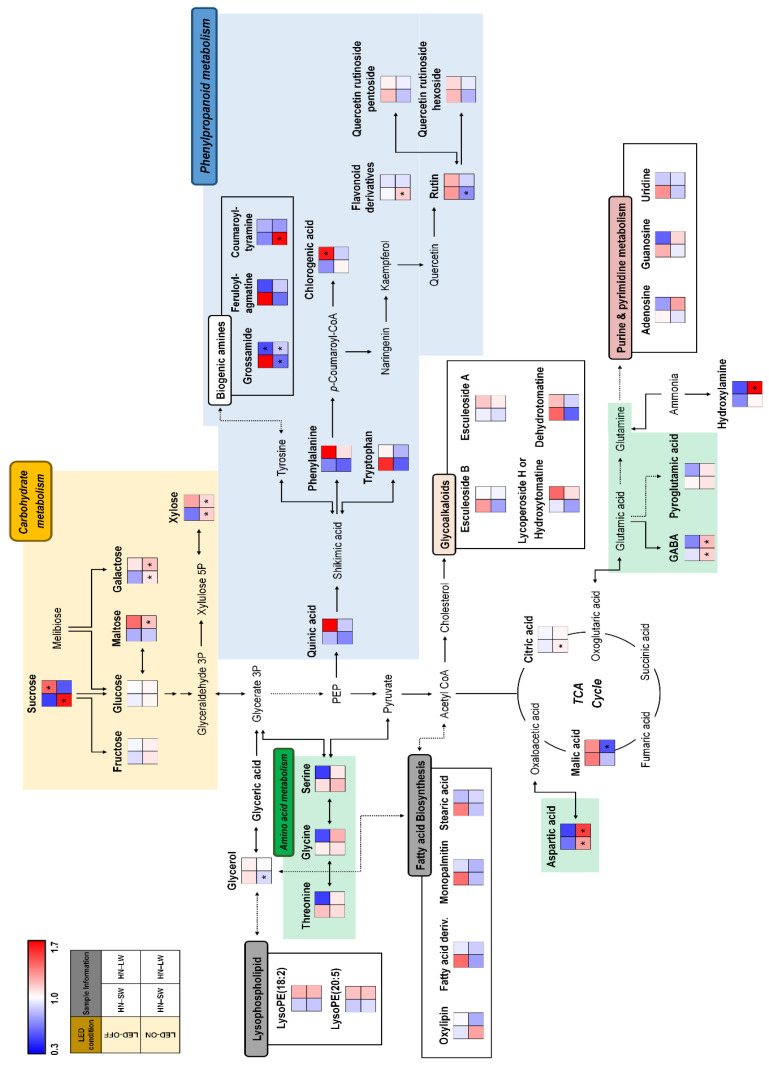
Constructed metabolic pathway for relative metabolite contents of tomato cultivated under different water and light condition for high N soil. The metabolic pathway was modified from the KEGG pathway database (https://www.genome.jp/kegg/pathway.html; URL accessed on 4 of June 2021). The colored squares (blue to red) indicate fold changes normalized by the average of each metabolite. * Significantly different metabolite from control (HN-SW-OFF) evaluated by *t*-test (*p* < 0.05).

Additionally, the relative levels of the discriminated metabolites in tomatoes cultivated with different water and red light conditions under high N soil were visualized in the corresponding metabolic pathway (Figure 5). The effect of red lighting was upregulating most of the sugars and sugar derivatives and carbohydrates metabolism-related metabolites. By contrast, phenylpropanoid metabolism-related metabolites including chlorogenic acid, quercetin rutinoside hexoside, quercetin rutinoside pentoside, and rutin were upregulated in red light-off conditions. Moreover, fatty acids, purine and pyrimidine, and polyamines related-metabolites were highly expressed in the HN-SW-OFF group. Intriguingly, glycoalkaloids and lipid metabolism-related metabolites were upregulated under low water conditions.

## 3. Discussion

Depending on the application of red lighting, lycopene content as the representative functional metabolite of tomato fruits was differently affected by nutrient and water supply in the glasshouse condition. The highest lycopene content was achieved by high N and limited water conditions in the absence of lighting. In terms of nutrient and water conditions, this was in line with the results of Wang and Xing [15]. They showed the highest lycopene content in tomato fruits grown under the highest nutrient supply × limited water supply in the greenhouse. De Pascale [21] also reported that tomatoes grown with salinized water of 4.4 dS m^−1^ in the field had greater lycopene content than with the non-salinized water of 0.5 dS m^−1^. By contrast, there was a report that either the lowest or medium level of N supply was needed for the highest lycopene content in tomatoes [12,13,14]. Low N supply has been suggested to enhance the lycopene contents of tomato fruits [11]. However, the current study did not. The effect of water was minor in this study. In the literature, water deficit either decreased lycopene [22] or increased [23,24]. The current study showed that in the absence of supplemental red light, lycopene contents were greatly affected by nutrient conditions. However, under the supplemental red light, the nutrient and water conditions did not play an important role in enhancing lycopene content. In the literature, light increased lycopene production. Light affected the lycopene synthesis rate, and it was increased by illuminating tomato plants during the fruit ripening [25]. Lycopene content was higher for tomatoes grown in the open field than under glass and plastic tunnels [26]. The effect of LED lighting on the lycopene production has also been known for detached tomato fruits as a post-harvest treatment, and the lycopene was higher after illumination of LED light than left in the darkness [27]. By the post-harvest treatment, the stimulation of lycopene production by red light was reversed by far-red light and this indicated the involvement of phytochromes in the mechanism [28]. In the current study, we did not observe any statistically significant increase in lycopene by supplemental light. Still, there was a tendency of increase by light in the low N condition, which is comparable to the literature. 

For primary metabolism, in the low N soil, red lighting increased some sugars (sucrose and maltose), and in the high N soil, red lighting increased sugars (xylose and galactose) in the current study. This is in line with Lu et al. [8], who showed that tomato plants grown under supplemental lighting for 28–55 days increased fruit sugar content compared to those treated for 10 days. They also reported higher fruit yield per plant in the longer supplemental lighting condition. In the current study, the low N soil greatly reduced amino acids. The decrease in amino acids by low N was in line with Gil et al. [1] for tomato fruits and leaves and roots of tomatoes and bell peppers [29,30]. Barneix and Causin [31] and Obata and Fernie [32] also reviewed that low nitrogen availability decreased amino acids in plant tissues. Deficit water supply is known to increase the concentrations of hexoses and organic acids on a fresh weight basis. However, they were not changing on the dry weight basis, indicating that the increase was related to the decrease in fruit water content [16]. Our study did not see the changes in hexoses and organic acids on the dry weight basis upon limited water supply. 

For secondary metabolism in our study, in the low N soil, the red lighting increased phenylpropanoids such as naringenin and guaiacol hexose pentose. In the standard N, red lighting did not significantly affect phenylpropanoids. In the high N soil, red lighting decreased most secondary metabolites, including phenylpropanoids, polyamines, and alkaloids. The phenylpropanoid pathway is affected by light quantity and quality in fruits, including tomatoes and berries [7]. The current study suggests that phenylpropanoids were differently regulated by light in different soil N conditions. Therefore, the effect of light should be evaluated while keeping the soil N condition in mind. In the current study, low N soil upregulated chlorogenic acid (phenolic acid) and low N is likely to induce a stress response, since phenylpropanoid is known to be upregulated as a response of plants against stress [33]. Polyamine metabolism is known to be upregulated by salinity and water deficit [34,35,36]. Therefore, the red lighting in the high N soil in our study seems to ameliorate the stress effect by high N. Polyamines are known to delay fruit ripening and softening, enhancing fruit quality by increasing fruit firmness and reducing fruit postharvest decay [37].

## 4. Materials and Methods 

### 4.1. Plant Materials, Soil Chemical Properties, and Supply Condition of Light, Nutrient, and Water 

Two-month-old tomato seedlings (*Solanum lycopersicum* L., cv. Super Dotaerang; indeterminate type, fresh market variety, globe, hybrid; Koregon Co., Ltd., Anseong, Korea; Solanaceae family) were transplanted into plastic pots containing 4.5 kg of soil in early March 2019 and were grown for 18 weeks at daily temperatures between 15–35 °C in a greenhouse in the National Institute of Agricultural Sciences, Rural Development Administration, Korea. Even though the temperature in the glasshouse fluctuated diurnally and seasonally, the heating and ventilating systems were operated to avoid extreme temperature conditions. The relative humidity in the glasshouse was not controlled. 

To vary the nutrient condition, three different conditions were employed, i.e., low N, standard N, and high N. For standard nutrient condition, soil with low electrical conductivity (EC; Table S1) was fed with the standard nutrient following the recommendations for tomato cultivation in greenhouse soil with low EC [38]. Three split applications of N and K fertilizer were conducted by topdressing (Table S2). For low N conditions, the three split fertilizer applications omitted N fertilizer (Table S2). For high N condition, high EC soil (Table S1) was fed with three split fertilizer applications, which were in accordance with the recommendations for tomato cultivation in greenhouse soil with high EC [38].

To analyze water supply effects, variations in water supply started at 12th week from the transplanting that was after fruit setting (BBCH code 71). Water was supplied either at −30 kPa for the sufficient supply or −80 kPa of soil water potential for the limited supply by reading tensiometers (Soilmoisture Equipment Corp., Santa Barbara, CA, USA). The tensiometer’s gypsum block, which senses the water potential, was placed at a depth of 0.07–0.13 m below the soil surface. There were 12 nutrient × water × light supply conditions. To improve the fruit setting, fully expanded flowers were treated with hormones (gibberellin and 4-chlorophenoxyacetic acid; BBCH code 63–65). Ripened fruits from 48 plants were harvested at similar ripening stages at 10:00 to avoid the diurnal changes in metabolites (four plants for each light × nutrient × water supply condition). Two to seven ripened tomato fruits (BBCH code 89) from each plant were harvested from 28 June to 24 July 2019. Before LED treatment (during the early stages of growth after transplantation), natural light was the only source of light. The LED structure was placed above the plants when the supplemental lighting started at the 12th week from the transplanting after fruit setting (BBCH code 71). The structure reduced the amount of natural light reaching the tomato plants, simulating the low light conditions in autumn and winter (PPFD: <500 μmol m^−2^ s^−1^). When the supplemental LED lighting was switched on, the plants were supplemented with a photosynthetic photon flux density (PPFD) of 90 μmol m^−2^ s^−1^ at the level of the plant canopy. The plants were irradiated with supplemental red LED lights (660 nm) for 6 h per day from 10:00 to 16:00. Plants without the supplemental LED lighting were placed under the same LED structure. However, LED lighting was not switched on. 

### 4.2. Sample Preparation and Extraction

Cultivated tomato fruits under different conditions were rinsed with distilled water and wiped before being stored at a deep freezer (‒80 °C). Frozen fruits samples were lyophilized and ground to powder using a mortar and pestle. Powdered samples were stored at ‒80 °C until metabolites extraction. Ground samples (100 mg) were extracted with 80% (*v*/*v*) methanol. The samples were sonicated for 10 min and then homogenized using Retsch MM 400 Mixer Mill (Retsch GmbH Co. Haan, Germany). After centrifugation for 10 min at 13,000 rpm and 4 °C (Gyrozen 1730R, Gyrozen Inc., Daejeon, Korea), the supernatants were filtered by polytetrafluoroethylene filter (Chromdisc, Daegu, Korea) and concentrated using a speed vacuum overnight. The final concentration of each analyzed sample was 20,000 ppm (20 mg/mL). 

### 4.3. Lycopene Analysis

Lycopene analysis was performed using the methods described by Kim et al. (2020) with few modifications [14]. Each powdered sample (200 mg) was extracted with 3 mL of the solvent mixture consisting of chloroform and DCM (2:1 *v/v*) using a shaker (Biofree, Seoul, Korea) at 100 rpm for 20 min and then added 1 mL of 1M sodium chloride solution. The extracts were centrifuged at 5000 rpm for 10 min at 4 °C to separate the layers. The filtered extracts were completely evaporated using nitrogen gas. The dried extracts were reconstituted with methanol and tert-Butyl methyl ether (MTBE) (3:2, *v/v*). The content of lycopene was analyzed by liquid chromatography diode array detection (LC-DAD) system. The Shimadzu LC system (UFLC NexeraX2 LC-30) was equipped with an autosampler (SIL-30AC), binary pump system (LC-30AD), and DAD detector (SPD-20A) were used for lycopene analysis. The lycopene extracts were analyzed based on the analytical methods described by Mun et al. (2021) [39]. The identification of lycopene was confirmed with its retention time and absorbance, which were compared to the standard lycopene compound analyzed under identical conditions.

### 4.4. GC-TOF-MS and UHPLC-LTQ-Orbitrap-MS Analysis

Primary metabolites were analyzed on Agilent 7890A system (Agilent, Santa Clara, CA, USA) equipped with Pegasus HT TOF-MS and L-PAL3 GC autosampler (LECO Corp., St.Joseph, MI, USA). GC-TOF-MS analysis was performed according to our previous study [14,39]. Before analysis, all dried extracts (100 μL) were derivatized through oximation and silylation reactions. The derivatized sample (1 μL) was injected into an instrument with a split ratio (1:20). The metabolites separation was performed on the RTX-5MS column (30 m × 0.25 mm; 0.25 μm pore size) with helium as the carrier gas at the constant flow rate of 1.5 mL/min. The analytical program and parameter setting for analysis were followed by our previous research [14,39]. The primary metabolites were identified using standard compounds compared with their retention time and MS fragments. Moreover, we confirmed the spectral database for primary metabolites available, such as the Human Metabolome Database (HMDB), National Institute of Standards and Technology (NIST; ver. 2.0, 2011, FairCom, Columbia, MO, USA), and Wiley 9 to improve accuracy.

A UHPLC-LTQ-Orbitrap-MS system coupled with a Vanquish binary pump H system (Thermo Fisher Scientific, Waltham, MA, USA) was used to investigate secondary metabolites. The analytical program for sample analysis was adopted for our previous study [18,39,40,41,42,43]. The secondary metabolite identification was performed based on various information comparing their retention time, MS spectrum, MS fragments, and elemental composition obtained from LC-MS data with an available database including published paper, in-house library, and web database or standard compounds analyzed under identical conditions. The sample analysis was performed for 5 biological replicates. To minimize potential the systematic errors, MS analysis was processed at random.

### 4.5. Data Processing and Multivariate Statistical Analysis

Raw data obtained from GC-TOF-MS and UHPLC-LTQ-Orbitrap-MS processing procedure and multivariate analysis were previously described by our previous studies [39]. The raw data files were converted to NetCDF (.cdf) file formats using LECO chromaTOF software (version 4.44, LECO Corp., St. Joseph, MI, USA) and Xcalibur software (Version 2.00, ThermoFisher Scientific, Waltham, MA, USA). After conversion, retention time correction, peak detection, and intensity, and mass data were determined using the MetAlign program (RIKILT-Institute of Food Safety, Wageningen, Netherlands). The multivariate analysis was performed using SIMCA-P+ (version 12.0, Umetrics, Umea, Sweden) to compare metabolites among the samples. The statistical variants for the partial least squares-discriminant analysis (PLS-DA) model were indicated using R^2^X and R^2^Y, representing the total sum of squares. In contrast, the fraction of total variation for X and Y components was signified by Q^2^. Moreover, the significance of the PLS-DA model was defined by analysis of variance testing of cross-validated predictive residuals (CV-ANOVA) in the SIMCA-P+ program. Significant differences in the non-targeted metabolite profiling were evaluated by student’s t-test using Predictive Analytics Software (PASW) Statistics 18 software (SPSS Inc., Chicago, IL, USA). Significant differences in the lycopene content were determined by ANOVA followed by Duncan’s multiple range test (*p* < 0.05). The normality of the data was not checked.

## 5. Conclusions

In summary, we examined the effects of nutrients × water × red light on the tomato fruit metabolites for plants grown under glasshouse conditions. The content of lycopene was highest in the condition of high N and limited water without a red light. In the absence of red light, the lycopene contents were greatly affected by nutrient and water conditions. Under the red light, the nutrient and water conditions did not play an important role in enhancing lycopene content. In the infertile soil, low N condition greatly reduced amino acids. The combination of red light to standard N affected little. However, the combination of red light to low N increased some sugars such as sucrose and maltose, and phenylpropanoids. The water supply resulted in minor effects. In the excessively fertile soil, red light greatly affected the metabolites, and water supply resulted in minor effects; red light increased many amino acids and sugars, including xylose and galactose. However, it decreased most of the secondary metabolites. We demonstrated that different nutrient conditions of soil resulted in different metabolites in tomato fruits. The effect of red light was clearly visible in the extreme nutrient conditions, i.e., low N and high N.

## Supplementary Materials

The following are available online at https://www.mdpi.com/article/10.3390/plants10071437/s1. Table S1: chemical properties of soils before transplanting tomato plants. Mean values of three replicates, Table S2: fertilizer applications for three different nutrient conditions, Table S3: differential primary metabolites identified by GC-TOF-MS in tomato fruits cultivated under varied lighting, nutrient, and water conditions, Table S4: differential secondary metabolites identified by UHPLC-LTQ-Orbitrap-MS in tomato fruits cultivated under varied lighting, nutrient, and water conditions, Figure S1: PCA score plots from GC-TOF-MS (A) and UHPLC-LTQ-Orbitrap-MS (B) analysis of tomato cultivated under different nutrient, water, and LED condition.
