# Supplementary material for: Combined Effects of Nutrients × Water × Light on Metabolite Composition in Tomato Fruits (Solanum Lycopersicum L.)
Source: Plants (Basel). 2021 Jul 14;10(7):1437. doi: 10.3390/plants10071437 (PMC8309447; doi:10.3390/plants10071437)
Supplement: Supplementary file 1 [file plants-10-01437-s001.zip › plants-1268208-sup.pdf]

Table S1. Chemical properties of soils before transplanting tomato plants. Mean values of three replicates.

Nutrient condition	pH (1:5)	EC (dS/m)	NO_3- N (mg/kg)	SOM (g/kg)	Av. P_2O_5 (mg/kg)	Ex. Cations ($cmol_c$/kg)			
						K	Ca	Mg	Na
Low EC soil	6.3	0.8	75	15	172	0.09	2.9	0.6	0.2
High EC soil	5.9	8.6	871	32	791	1.69	13.3	5.0	1.8

Table S2. Fertilizer applications for three different nutrient conditions

Fertilizer application	Time	Fertilization for low EC soil (g/kg soil)				Fertilization for high EC soil (g/kg soil)
		Low N		Standard N		High N
		N	K	N	K	K
1st supply	1.5 months after transplant	0	0.14	0.05	0.14	0.05
2nd supply	2 months after transplant	0	0.14	0.05	0.14	0.05
3rd supply	3.5 months after transplant	0	0.14	0.05	0.14	0.05

Table S3. Differential primary metabolites identified by GC-TOF-MS in tomato fruits cultivated under varied lighting, nutrient, and water conditions

No.	Ret (min) [a]	Metabolites [b]	Unique Mass (*m/z*)	MS Fragment pattern (*m/z*)	ID [c]
Amino acids					
1	7.26	Isoleucine *	158	73, 57, 117, 130, 147, 158, 132, 219, 116	STD/MS
2	7.39	Glycine	174	73, 174, 86, 147, 107, 100, 133, 175, 118	STD/MS
3	7.90	Serine	204	73, 100, 204, 188, 147, 59, 218, 133	STD/MS
4	8.14	Threonine	219	73, 57, 117, 101, 147, 219, 218, 100	STD/MS
5	8.48	β-Alanine *	248	73, 160, 130, 116, 174, 131, 161, 248	STD/MS
6	9.28	Aspartic acid	232	73, 232, 100, 84, 147, 117, 218, 233, 133	STD/MS
7	9.35	Pyroglutamic acid	156	73, 156, 174, 147, 157, 158, 230, 258	STD/MS
8	9.36	GABA	174	73, 174, 84, 147, 86, 175, 157, 100	STD/MS
9	10.16	Phenylalanine	192	73, 218, 100, 192, 147, 84, 59, 219, 130	STD/MS
10	14.14	Tryptophan	202	73, 75, 117, 217, 202, 129, 130	STD/MS
Organic acids					
11	7.43	Succinic acid *	247	73, 147, 107, 77, 55, 69, 84, 247, 256	STD/MS
12	9.02	Malic acid	233	73, 147, 133, 55, 101, 233, 148, 117	STD/MS
13	11.61	Citric acid	273	73, 147, 273, 211, 149, 133, 129	STD/MS
Sugars and sugar derivatives					
14	7.07	Glycerol	205	73, 147, 117, 103, 133, 205, 148, 218	STD/MS
15	7.62	Glyceric acid *	189	73, 147, 189, 133, 103, 117, 130, 148	STD/MS
16	10.42	Xylose	103	73, 103, 217, 147, 133, 117, 307, 189	STD/MS
17	10.94	Ribose *	205	73, 117, 147, 89, 59, 133, 103, 205, 217	STD/MS
18	12.10	Fructose	103	73, 103, 217, 147, 307, 133, 218, 117	STD/MS
19	12.28	Galactose	160	73, 147, 160, 205, 103, 217, 319, 117	STD/MS
20	12.37	Glucose	103	73, 147, 103, 160, 205, 217, 319, 129	STD/MS
21	12.53	Glucoronic acid *	333	73, 147, 103, 160, 217, 333, 133, 129	STD/MS
22	13.44	myo-Inositol *	217	73, 147, 217, 191, 103, 129, 133, 305	STD/MS
23	14.82	Glucose 6-phosphate *	299	73, 147, 103, 299, 129, 387, 160, 217	STD/MS
24	15.35	myo-Inositol phophate *	299	73, 147, 217, 299, 318, 103, 315, 133	MS
25	16.51	Sucrose	361	73, 147, 103, 217, 129, 169, 361, 271	STD/MS
26	17.03	Maltose	361	73, 147, 103, 361, 217, 191, 129, 204	STD/MS
Fatty acids and lipids					
27	12.97	Palmitic acid *	117	73, 147, 75, 117, 129, 133, 132, 313	STD/MS
28	14.14	Stearic acid **	117	73, 75, 117, 103, 129, 217, 132, 341	STD/MS
29	16.04	Monopalmitin	371	73, 147, 75, 57, 129, 103, 117, 133, 371	STD/MS
30	16.98	Fatty acid derivatives **	129	73, 147, 57, 129, 117, 101, 133, 203, 399	MS
Nucleosides and nucleotides					
31	7.11	Adenosine 5'-diphosphate *	299	73, 299, 133, 193, 211, 300, 207, 191	STD/MS
32	15.49	Uridine	169	73, 217, 130, 174, 147, 169, 129, 259	STD/MS
33	16.40	Adenosine	230	73, 230, 236, 103, 147, 217, 245, 192	STD/MS
34	17.09	Guanosine	324	73, 217, 147, 324, 129, 245, 230, 169	STD/MS

Others					
35	5.41	Hydroxylamine **	146	73, 133, 146, 119, 59, 86, 147, 130, 100	STD/MS
36	11.92	Quinic acid	345	73, 147, 345, 255, 103, 133, 191, 148	STD/MS
Unknowns					
37	6.46	N.I. 1 **	228	73, 69, 58, 110, 77, 228, 134, 184, 127	– [d]
38	6.92	N.I. 2 *	116	73, 116, 132, 75, 57, 74, 103, 117, 147	–
39	8.45	N.I. 3	160	73, 75, 160, 130, 117, 147, 100, 161, 131	–
40	9.24	N.I. 4 **	83	84, 73, 158, 85, 157, 186, 140, 116, 61	–
41	9.55	N.I. 5 **	120	120, 73, 75, 84, 146, 130, 103, 174	–
42	11.84	N.I. 6 *	173	73, 55, 173, 157, 147, 129, 174, 316	–
43	15.67	N.I. 7 **	103	73, 103, 147, 217, 68, 117, 129, 446	–
44	15.81	N.I. 8	446	73, 103, 147, 156, 68, 217, 117, 446	–

[a] Retention time.; [b] Primary metabolites selected by VIP value (> 1.0) based on PLS-DA model.; [c] Identification. STD/MS, comparing with standard compounds analyzed under same condition and mass spectrum comparison with HMDB, NIST, and wiley 9 database.; [d] not detected.; * Only selected metabolites in standard and low N soil condition; ** Only selected metabolites in high N soil condition.

Table S4. Differential secondary metabolites identified by UHPLC-LTQ-Orbitrap-MS in tomato fruits cultivated under varied lighting, nutrient, and water conditions

No.	Ret (min)	Tentative Identification [a]	Identified ion (*m/z*)		MS^n fragment pattern (*m/z*) [b]	Elemental composition	Delta ppm	Reference
			$[M+H]^+$	$[M–H]^–$				
Phenylpropanoids								
1	2.09	Caffeic acid glucose *	–	341.0904	341> 281, 179> 135	C15H17O9 (–)	7.490	REF [24]
2	3.66	Chlologenic acid	355.1014	353.0921	353> 191, 179, 173, 135	C16H17O9 (–)	-2.756	REF [22]
3	3.78	Quercetin rutinoside hexoside	773.2116	771.2064	771> 609, 463> 301> 271, 255, 179	C33H39O21 (–)	-2.683	REF [22]
4	3.84	Guaiacol hexose pentose *	441.1354 [c]	463.1510 [d]	463> 417, 293> 275, 233, 191, 149	C18H26O11Na (+)	-3.066	REF [25]
5	4.43	Quercetin rutinoside pentoside **	743.2014	741.1984	741> 609, 300> 271, 255	C32H39O20 (+)	-2.664	REF [22]
6	4.60	Rutin	611.1593	609.1555	609>301> 271, 255, 179, 151	C27H31O16 (+)	-2.227	REF [22]
7	4.84	Kaempferol rutinoside *	595.1645	593.1558	593> 525, 285> 257, 229, 163	C27H31O15 (+)	-2.111	REF [22]
8	5.36	Tricaffeoylquinic acid *	679.2957	677.3019	677> 353> 335, 191, 173	C34H29O15 (–)	-2.811	REF [22]
9	5.61	Flavonoids derivatives	255.0641	253.0568	253> 224, 209, 197> 165, 153, 139	C15H11O4 (+)	-4.726	LIB [e]
10	6.15	Naringenin *	273.0752	271.0635	271> 225, 177, 151> 107	C15H13O5 (+)	-1.831	REF [S2]
Polyamines								
11	4.01	Feruloylagmatine **	307.1754	305.1656	(+) 307> 247, 230, 177, 145	C15H23N4O3 (+)	-3.311	REF [23]
12	5.51	Coumaroyl tyramine **	284.1272	282.1155	282> 240, 177, 162, 145, 119	C17H18NO3 (+)	-3.238	REF [23]
13	6.63	Grossamide	625.2519	623.2587	623> 460> 297> 282, 175	C36H37N2O8 (+)	-4.082	WebDB [f]
Alkaloids								
14	4.31	Esculeoside B	1228.5942	1272.6053 [e]	1272> 1226> 1094, 932, 770, 608	C56H94NO28 (+)	-1.178	REF [24]
15	4.77	Esculeoside A	1270.6033	1268.6061	1268> 1136, 974, 932> 752, 590	C58H96NO29 (+)	-2.347	REF [22]
16	4.83	Lycoperoside H or Hydroxytomatine	1050.5461	1094.5671 [e]	– [h]	C50H84NO22 (+)	-1.722	REF [24]
17	5.36	Dehydrotomatine **	1032.5355	1076.5585 [e]	–	C50H82NO21 (+)	-1.806	REF [26]
18	5.44	Tomatine *	1034.5493	1078.5707 [e]	–	C50H84NO21 (+)	-3.591	REF [26]
Oxylipins and lipids								

19	6.19	9,12,13-TriHODE *	351.2137 [c]	327.2215	327> 309, 291, 229, 211, 171> 153	C18H32O5Na (+)	-1.467	REF [22]
20	7.85	LysoPC(18:3) *	518.3220	562.3192 [d]	562> 502, 431> 277	C26H49NO7P (+)	-4.043	REF [22]
21	8.14	LysoPE(18:2)	478.2909	476.2819	476> 402, 384> 357	C23H45NO7P (+)	-3.441	REF [22]
22	8.14	LysoPE(20:5)	500.2732	544.2698 [d]	544> 498, 476> 279	C25H43NO7P (+)	-8.007	HMDB [g]
23	8.23	LysoPC(18:2) *	520.3379	564.3346 [d]	564> 504, 391> 279, 242	C26H51NO7P (+)	-3.605	REF [22]
24	8.56	LysoPE(16:0) *	454.2913	452.2807	452> 391, 255> 237	C21H45NO7P (+)	-3.292	REF [22]
25	8.64	LysoPC(16:0) *	496.3377	540.3355 [d]	540> 480, 391> 255, 224	C24H51NO7P (+)	-4.142	REF [22]
26	8.64	Oxylipin **	311.2205	309.1771	309> 291, 273, 185, 171, 155	C18H31O4 (+)	-3.843	REF [27]
27	8.72	LysoPC(18:1) *	522.3533	566.3500 [d]	566> 506, 415> 281, 242	C26H53NO7P (+)	-3.993	REF [23]
28	8.86	LysoPA(18:2) *	435.2491	433.2386	433> 415, 365, 153> 97, 79	C21H40O7P (+)	-3.461	REF [23]
29	9.50	LysoPC(18:0) *	524.3684	568.3646 [d]	568> 508, 421, 391> 283, 224	C26H55NO7P (+)	-5.065	REF [23]
Others								
30	3.80	Benzyl alcohol hexose pentose *	425.1406 [c]	447.1559 [d]	447> 401> 269, 233, 161	C18H26O10Na (+)	-2.795	REF [25]
Unknowns								
31	1.01	N.I. 1	328.1378	326.1264	326> 308, 278, 236, 206, 164	–	–	–
32	3.99	N.I. 2	469.1666 [c]	491.1823 [d]	491> 445> 323, 221, 179	–	–	–
33	4.03	N.I. 3	409.1822 [c]	431.1957 [d]	431> 385> 223, 205, 161, 153	–	–	–
34	4.27	N.I. 4	439.1559 [c]	461.1758 [d]	461> 415, 311> 251, 191, 149	–	–	–
35	6.99	N.I. 5 **	449.1473	447.1385	–	–	–	–

[a] Secondary metabolites selected by VIP value (> 1.0) based on PLS-DA model.; [b] MS^n fragment patterns detected in negative ion mode.; [c] $[M+Na]^+$; [d] $[M+HCOOH–H]^-$; [e] In house library; [f] Web database (KNApSAcK core system; http://www.knapsackfamily.com/knapsack_core/top.php); [g] The Human Metabolome Database (HMDB; https://hmdb.ca/); [h] Not detected.; * Only selected metabolites in standard and low N soil condition; ** Only selected metabolites in high N soil condition.

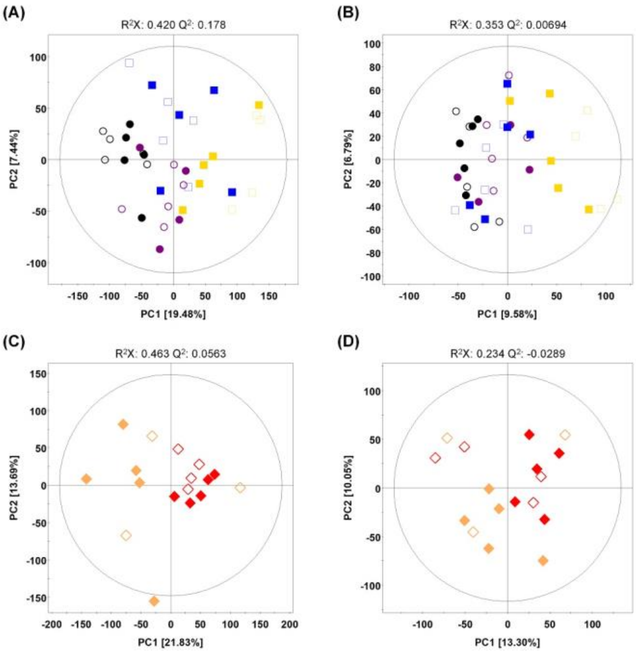

Figure S1. PCA score plots from GC-TOF-MS **(A)** and UHPLC-LTQ-Orbitrap-MS **(B)** analysis of tomato cultivated under different nutrient, water, and light condition. Symbol, SN-SW-OFF (■); SN-LW-OFF (□); LN-SW-OFF (●); LN-LW-OFF (○); SN-SW-ON (■); SN-LW-ON (□); LN-SW-ON (●); LN-LW-ON (○). SN, standard soil; SW, sufficient water; OFF, red light off; LN, Low-N; LW, limited water; ON, red light on. PCA score plots from GC-TOF-MS **(C)** and UHPLC-LTQ-Orbitrap-MS **(D)** analysis of tomato cultivated under water, and light condition for high N soil. Symbol, HN-SW-OFF (◆); HN-LW-OFF (◇); HN-SW-ON (◆); HN-LW-ON (◇). HN, high N soil; SW, sufficient water; OFF, red light off; LW, limited water; ON, red light on.
